# Tunable band-gap structure and gap solitons in the generalized Gross-Pitaevskii equation with a periodic potential

**DOI:** 10.1038/s41598-018-19756-6

**Published:** 2018-01-22

**Authors:** Changming Huang, Liangwei Dong

**Affiliations:** Department of Physics, Shaanxi University of Science & Technology, Xi’an, 710021 China

## Abstract

The tunable band-gap structure is fundamentally important in the dynamics of both linear and nonlinear modes trapped in a lattice because Bloch modes can only exist in the bands of the periodic system and nonlinear modes associating with them are usually confined to the gaps. We reveal that when a momentum operator is introduced into the Gross-Pitaevskii equation (GPE), the bandgap spectra of the periodic system can be shifted upward parabolically by the growth of the constant momentum coefficient. During this process, the band edges become asymmetric, in sharp contrast to the standard GPE with an external periodic potential. Extended complex Bloch modes with asymmetric profiles can be derived by applying a phase transformation to the symmetric profiles. We find that the inherent parity-time symmetry of the complex system is never broken with increasing momentum coefficient. Under repulsive interactions, solitons with different numbers of peaks bifurcating from the band edges are found in finite gaps. We also address the existence of embedded solitons in the generalized two-dimensional GPE. Linear stability analysis corroborated by direct evolution simulations demonstrates that multi-peaked solitons are almost completely stable in their entire existence domains.

## Introduction

The Bloch band is crucial to our understanding of periodic systems. Its underlying physics is the Bragg reflection occurring at the edges of the Brillouin zone. The interplay between spatial periodicity and nonlinear dynamical evolution excites great interest in many-body effects. The experimental realization of optical lattices is thus important in almost all physical fields^1^. In nonlinear science, optical lattices provide an effective means for the guidance, management, and control of the dynamics of objects described by wave functions, including solitary waves in Bose-Einstein condensates (BECs)^2,3^, nonlinear optics^4,5^, and nonlinear polariton topological insulators^6–8^. According to the Floquet-Bloch theorem, the band-gap spectra of a periodic system determine the forms and existence domains of solitary waves associating with the corresponding linear Bloch modes^4,5^.

Atomic gases trapped in external potentials provide a versatile tool for simulating phenomena predicted in other branches of physics. The observation of spin-orbit coupling (SOC) effects in BECs^9,10^ and fermionic gases^11^ attracts unprecedented attention these days in diverse areas of science such as matter waves, solid-state physics, acoustics, and photonics (see, e.g.^6,7^, for recent reviews). Recent progress in SOC-BECs significantly enriches the possibilities that many fundamental phenomena can be emulated or modeled by cold atom systems. Some unique characteristics, such as *Zitterbewegung*^12^, the quantum spin Hall effect^13^, topological quantum phase transitions^14^, and chiral topological superfluid phases^15^, have been reported. The most striking and interesting phenomenon relating to SOC is the topological insulators^6,7^ and the relevant nonlinear modes^8,16–19^ trapped in optical lattices. They are described by a system of coupled GPEs or nonlinear Shrödinger equations for the spinor polariton wave function^7,8,18^.

The intrinsic nonlinearity, originating from interatomic interactions, supports solitary waves in BECs confined in harmonic potentials or optical lattices^2,3^. Diverse types of localized nonlinear excitations were predicted to be supported by SOC condensates. For example, coherent structures in the form of gap^17^, dark^20^, and bright^21^ solitons have been found in SOC-BECs. Topological structures such as skyrmions^22^, vortices^23^, and Dirac monopoles^24^ were also uncovered.

While most current studies focusing on the topologically protected unidirectional edge states in honeycomb lattices are based on the combination of SOC and Zeeman splitting, little attention has been paid to the dynamics of nonlinear excitations in a scalar GPE with a momentum operator, which can be reduced from the coupled GPEs.operator *iγ*(*x*)∂/∂*x* is introduced into the system, the generalized Hamiltonian operator becomes ${\mathcal H} =\frac{1}{2}{\partial }^{2}/\partial {x}^{2}+i\gamma (x)\partial /\partial x+V(x)$. Stable solitons were found in nonlinear systems described by the generalized Hamiltonian operator with several types of external potentials, e.g., the rotating harmonic-plus-quartic potential^25,26^, anharmonic^25^ and two narrow Gaussian barrier^27^ potentials. More recently, Yan *et al*. reported the propagation dynamics of solitons in the generalized GPE with a localized parity-time (${\mathscr{P}}{\mathscr{T}}$) symmetric Scarff-II potential^28^.

Thus far, the bandgap structure is usually adjusted by changing the lattice depth or frequency. For a fixed lattice, other methods for the realization of tunable bandgap spectra have not yet been proposed. Moreover, the evolution of matter-wave gap solitons in the generalized scalar GPE with a momentum operator modulated by a periodic lattice has not yet been explored. In this paper, we will address two closely related problems. First, we reveal that the bandgap structure of a system with a fixed lattice can be shifted parabolically by the growth of the constant momentum coefficient. This feature offers us a new way, instead of using the lattice depth or frequency, to tune the bandgap spectra of a periodic system. Second, we find several families of nonlinear waves with different numbers of peaks. Such solitons bifurcate from the complex Bloch modes at the band edges, and their peaks are of the same value, analogous to the truncated-Bloch-wave^29^ and multi-peaked gap solitons in ${\mathscr{P}}{\mathscr{T}}$-symmetric lattices^30^. In addition, we demonstrate the existence and dynamics of two-dimensional (2D) in-band solitons in the system with a quasi-one-dimensional (1D) lattice. Linear stability analysis results demonstrate that different families of solitons are stable in almost their whole existence domains.

## Theoretical Model

We start our analysis from the 1D coupled Gross-Pitaevskii equations or nonlinear Shrödinger equations:^18,19^

1 $$i\frac{\partial {\rm{\Psi }}}{\partial t}=\frac{1}{2}{(\frac{1}{i}\frac{\partial }{\partial x}-\kappa (x){\sigma }_{1})}^{2}{\rm{\Psi }}+\frac{{\rm{\Omega }}}{2}{\sigma }_{3}{\rm{\Psi }}-g({{\rm{\Psi }}}^{\dagger }{\rm{\Psi }}){\rm{\Psi }}-V^{\prime} (x){\rm{\Psi }},$$

where the spinor Ψ = (*ψ*_1_, *ψ*_2_)^*T*^ describes the quasi-1D spin-orbit-coupled Bose-Einstein condensates trapped in an optical potential *V*′(*x*), *σ*_1,3_ are the Pauli matrices, and Ω denotes the Zeeman splitting. *κ*(*x*) = (*d*)/(*dx*)*K*(*x*), with *K*(*x*) being a phase modulation. In the absence of an external potential, Eq. (1) is exactly integrable if either Zeeman splitting or spin-orbit coupling is considered alone^18^.

By setting *ψ*_1_ = *ψ*_2_ = *ψ*, *κ*(*x*) = 2*γ*(*x*), $V^{\prime} (x)=V(x)+\frac{1}{2}[i{k}_{x}(x)+{k}^{2}(x)]$, and ignoring the Zeeman splitting (Ω = 0), one obtains a generalized dimensionless scalar GPE:

2 $$i\frac{\partial \psi }{\partial t}=-\frac{1}{2}\frac{{\partial }^{2}\psi }{\partial {x}^{2}}-i\gamma (x)\frac{\partial \psi }{\partial x}-V(x)\psi -g|\psi {|}^{2}\psi .$$

Here, *ψ*(*x*, *t*) is a complex condensate wave function, $i\gamma (x)\frac{\partial }{\partial x}$ is the so-called momentum operator with *γ*(*x*) being the coefficient^28^, *V*(*x*) is an external trapping potential and *g* = ±1 stand for the attractive and repulsive nonlinear interactions. Eq. (2) describes a BEC cloud loaded into an optical lattice in the mean-field approach. This model equation is made dimensionless by using the characteristic scales of the lattice, length *a*_*L*_ = *d*/*π*, energy ${E}_{rec}={\hslash }^{2}/2\,m{a}_{l}^{2}$ and time ${\omega }_{L}^{-1}=\hslash /{E}_{rec}$, where d is the lattice period and m the mass of the trapped atoms. The wave function is in units of $1/\sqrt{8\pi {a}_{L}^{2}|{a}_{s}|}$ where *a*_*s*_ is the scattering length of the condensed atoms.

When a real harmonic lattice $V(x)=p\,{\cos }^{2}(x)$ is considered, the system becomes nonintegrable and must be solved numerically. We are interested in stationary localized solutions that can be searched by inserting the form of a plane wave solution Ψ(*x*, *t*) = *ϕ*(*x*)exp(*ibt*) into Eq. (2). Here, *b* is the chemical potential, and *ϕ* = *ϕ*_*r*_ + *iϕ*_*i*_ is the complex soliton profile, including real and imaginary components. The derived nonlinear differential equation can be written as

3 $$\frac{1}{2}\frac{{\partial }^{2}\varphi }{\partial {x}^{2}}+i\gamma (x)\frac{\partial \varphi }{\partial x}-b\varphi +V(x)\varphi +g|\varphi {|}^{2}\varphi =0.$$

The power of solitary waves can be defined as $U={\int }_{-\infty }^{+\infty }|\varphi (x{)|}^{2}dx$, and the Hamiltonian is $H={\int }_{-\infty }^{+\infty }[\frac{1}{2}|\frac{\partial \varphi }{\partial x}{|}^{2}-i\frac{\gamma }{2}$ $({\varphi }^{\ast }\frac{\partial \varphi }{\partial x}-\varphi \frac{\partial {\varphi }^{\ast }}{\partial x})-V(x)|\varphi {|}^{2}-\frac{1}{2}g|\varphi {|}^{4}]dx$. In the following discussion, for simplicity, we set *γ*(*x*) = *γ* and $V(x)=6\,{cos}^{2}(x)$, and we focus on the repulsive nonlinearity *g* ≡ −1. Stationary solutions of nonlinear modes can be solved numerically either by the relaxation method or by the Newton-conjugate-gradient method^31^.

## Tunable bandgap structure and the corresponding Bloch modes

To understand the basic properties of the guided nonlinear modes, it is instructive to consider the Floquet-Bloch spectrum of the corresponding linear system. The spectrum of the system described by Eq. (2) is determined by the linear eigenvalue problem *Lϕ*(*x*) = *bϕ*(*x*), with $L=\frac{1}{2}\frac{{\partial }^{2}}{\partial {x}^{2}}+i\gamma \frac{\partial }{\partial x}+V(x)$, where *b* and *ϕ*(*x*) are the eigenvalue and eigenfunction, respectively. For the standard GPE with *γ* = 0, the linear system degenerates to the usual Hamiltonian operator *L*_0_*ϕ*_0_(*x*) = *b*_0_*ϕ*_0_(*x*), which admits an entirely real eigenvalue and eigenfunction. Applying the invertible transformation *ϕ*_0_(*x*) = *ϕ*(*x*)*exp*(*iγx*), one immediately finds that *b* = *b*_0_ + *γ*^2^/2 holds for all nonzero momentum coefficients.

In particular, due to the presence of the imaginary momentum operator, although the eigenfunction becomes complex for a real *V*(*x*), the spectrum remains real for any real *γ*(*x*). Substituting *ψ*(*x*, *t*) = *ϕ*(*x*)exp(*ikt*) into Eq. (2), where *k*∈[−1, 1] is the wave number and *ϕ*(*x*) = *ϕ*_*r*_(*x*) + *iϕ*_*i*_(*x*) is the complex Bloch mode, after dropping the nonlinear term, one obtains

4 $$\frac{1}{2}\frac{{d}^{2}\varphi }{d{x}^{2}}+2ik\frac{d\varphi }{dx}-i\gamma \frac{d\varphi }{dx}-{k}^{2}\varphi +V\varphi =b\varphi \mathrm{.}$$

The spectra of the system can be solved numerically by the plane-wave expansion method^32^.

**Figure 1:**
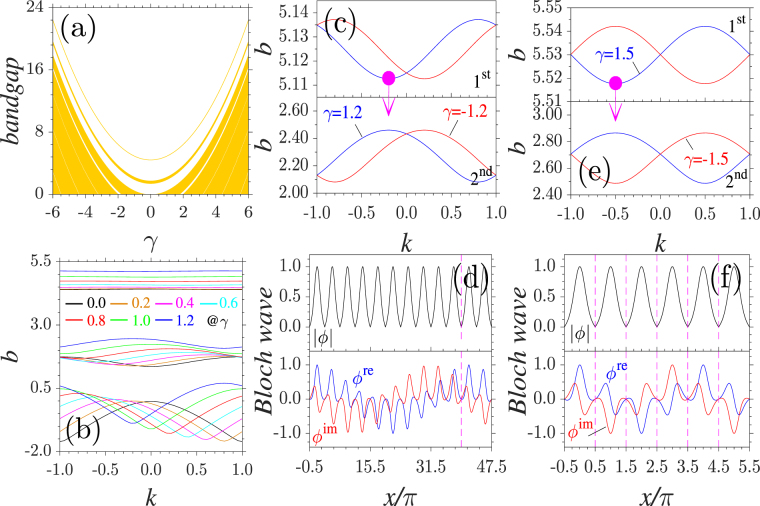
Bandgap structure. (**a**) Band-gap structure of a fixed periodic lattice. Bands are marked with yellow; gaps are shown as white. (**b,c,e**) The detailed band spectra versus Bloch wave number *k*. The lines with the same colour share the same *γ* and *δγ* = 0.2 in (**b**). (**d,f**) Complex Bloch modes at the lower edge of the first band marked by solid circles in (**c**) and (**e**). Extended Bloch modes residing in different lattice channels are also shown.

To illustrate the role of the momentum operator, we plot the bandgap structure versus the momentum coefficient *γ* in Fig. 1(a). At *γ* = 0, the spectrum of the linear system with a periodic lattice is composed of an infinite number of bands and gaps sandwiched between the neighbouring bands. As predicted by the above analysis, the bands and gaps are shifted towards the direction of the semi-infinite gap with the growth of *γ*. Each band and each gap move upward in a parabolic manner. For example, at the lower edge of the first band, *b* = 4.39 at *γ* = 0 and *b* = 22.39 at *γ* = 6. Obviously, the relationship *b*_*γ*=6_ = *b*_*γ*=0_ + *γ*^2^/2 holds.

This property offers us an effective method for the realization of the tunable bandgap structures of a fixed lattice. In other words, instead of the lattice depth, one can utilize the momentum coefficient to change the spectra. Concretely, *b* = 5 belongs to the semi-infinite gap at *γ* = 0 and the first gap at *γ* = 2. When *γ* = 2.8, the same *b* resides in the second gap. This allows one to study the dynamics of different families of nonlinear modes at the same *b* value but bifurcating from the edges of different bands.

The band spectra versus *k* in the first Brillouin zone at *γ* = 0 to 1.2 are shown in Fig. 1(b). With the growth of *γ*, the band edges become asymmetric, which is in sharp contrast to conventional systems with a periodic lattice^29^ or a ${\mathscr{P}}{\mathscr{T}}$ symmetric lattice^33^. A specific case of band spectra at *γ* = ±1.2 is displayed in Fig. 1(c). At *γ* = 1.2, the lower and upper edges of the first band locate at *k*_low_ = −0.2 and *k*_upp_ = 0.8, respectively. The wave numbers of the lower and upper edges of the second band are opposite to those of the first band. The band edges at *γ* = −1.2 are symmetric with those at *γ* = 1.2, which results in the fact that the edges of the first two bands locate at *k* = 0.2 and −0.8. At *γ* = ±1.2, *δb*_1st_ = 0.024 and *δb*_2nd_ = 0.3780, which exactly equals the width of the first and second bands at *γ* = 0. This demonstrates that the bands or the open gaps remain the same width when they are shifted by the increase in *γ*.

Due to the presence of the imaginary momentum operator, the eigenmodes are now complex [Fig. 1(d)]. The Bloch mode at the lower edge of the first band with *γ* = 1.2 includes two components with different symmetries. The real part is even symmetric, and the imaginary part is odd symmetric. However, by applying a constant phase transformation in the form of *exp*(*iθ*) with *θ* = (*k*_low_*π*/*d*)*d* = 0.2*π* to the Bloch mode at *k*_low_, one derives a series of extended Bloch modes covering many lattice periodicities. While the real and imaginary parts of the extended Bloch modes are no longer symmetric, their moduli remain even symmetric. After *n* = 2*π*/|*θ*| = 10 consecutive transformations, the mode can be recovered to the original mode. Reversely, any asymmetric Bloch mode can be transformed as a symmetric Bloch mode with even real and odd imaginary parts.

In general, the action of the parity operator $\hat{P}$ is defined by the relations $\hat{p}\to -\hat{p},\hat{x}\to -\hat{x}$, where $\hat{p}$ and $\hat{x}$ are the momentum and position operators, respectively. The time operator $\hat{T}$ is defined by $\hat{p}\to -\hat{p},\hat{x}\to \hat{x}$, and *i* → −*i*. Thus, $\hat{T}\hat{H}=\hat{T}[\frac{1}{2}{\hat{p}}^{2}+\gamma \hat{p}+V(x)]=\frac{1}{2}{\hat{p}}^{2}-\gamma \hat{p}+{V}^{\ast }(x)$ and $\hat{P}\hat{T}\hat{H}=\frac{1}{2}{\hat{p}}^{2}+\gamma \hat{p}+{V}^{\ast }(-x)$. To obtain $\hat{P}\hat{T}\hat{H}=\hat{H}$, one only needs *V*(*x*) = V^*^(−*x*). Obviously, the linear version of Eq. (2) respects the ${\mathscr{P}}{\mathscr{T}}$ symmetry because the even real potential *V*(*x*) satisfies the condition. A unique feature that we should stress is that with increasing *γ*, the eigenvalue of the system never becomes complex, which indicates that the ${\mathscr{P}}{\mathscr{T}}$ symmetry in our system is unbreakable. Note that the only two known examples of unbreakable ${\mathscr{P}}{\mathscr{T}}$ symmetry were found in inhomogeneous defocusing nonlinearities with an antisymmetric localized^34^ or periodic gain and loss^35^.

The detailed band spectra at *γ* = ±1.5 and the corresponding Bloch modes are illustrated in Fig. 1(e) and (f), respectively. Now, the band edges reside at *k* = ±0.5 for both bands, and the band edges are antisymmetric about *k* = 0. The *b* value at the marker in Fig. 1(c) is 5.113, and the *b* value at the marker in Fig. 1(e) is 5.518. One finds that *δb* = 0.405, which exactly equals 1.5^2^/2 − 1.2^2^/2. This proves again that the above derived relationship *b* = *b*_0_ + *γ*^2^/2 holds for all *γ*. Similar to the cases of *γ* = ±1.2, the extended Bloch modes covering other lattice channels can be obtained by applying a constant phase transformation in the form of *exp*(*iθ*) with *θ* = (*k*_low_*π*/*d*)*d* = 0.5*π* to the Bloch mode at *k*_low_. However, the consecutive transformations do not involve asymmetric modes. Specifically, the components of the extended Bloch modes satisfy either even or odd symmetries [Fig. 1(f)]. The times for recovering the Bloch mode at *k* = −0.5 is *n* = 2*π*/|*θ*| = 4.

We should stress that at *γ* = 0, the extended Bloch modes with other forms cannot be obtained by a constant phase transformation. All the Bloch modes in different lattice channels are identical and cannot be distinguished. It is the nonzero *γ* that affords the emergence of extended asymmetric Bloch modes and the asymmetric nonlinear excitations originating from them.

## Families of solitons bifurcating from complex linear Bloch modes

**Figure 2:**
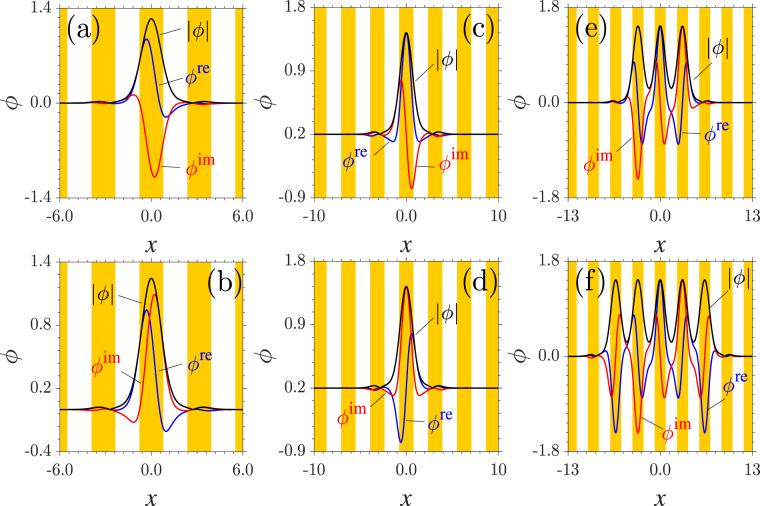
Soliton profiles. (**a**,**b**) Asymmetric solitons originating from the asymmetric extended Bloch modes shown in Fig. 1(d). (**c**,**d**) Profiles of symmetric solitons bifurcating from Bloch modes residing in the first and fourth lattice sites [see Fig. 1(f)]. (**e**,**f**) Profiles of three- and five-peaked gap solitons. *γ* = 1.2 in (**a,b**) and 1.5 in (**c–f**). *b* = 4.0 in all the panels.

Now, we consider the nonlinear modes bifurcating from the Bloch modes at the lower edge of the first band. Families of stationary solutions are found in the first gap [Fig. 2]. Solitons can bifurcate from any extended Bloch modes shown in Fig. 1(d). At *γ* = 1.2, both the real and imaginary parts of solitons originating from the asymmetric extended Bloch modes exhibit strong asymmetries [Fig. 2(a) and (b)]. It is worth mentioning that the solutions with ${\mathscr{P}}{\mathscr{T}}$ symmetries (i.e., an even real part and an odd imaginary part) associating with the Bloch modes at the lower edge of the first band [see the first Bloch mode shown in Fig. 1(d)] can be obtained by applying a phase transformation to the asymmetric modes. When *γ* = 1.5, the lower edge of the first band is at *k*_low_ = −0.5. The real and imaginary parts of the solitons at *b* = 4.0 originating from the Bloch mode in the region *x* ∈ [−0.5*d*,0.5*d*] (*d* is the periodicity of the potential) respect even and odd symmetries, respectively [Fig. 2(c)]. The symmetries are reversed when a soliton bifurcates from the Bloch mode in the region *x* ∈ [2.5*d*, 3.5*d*] [Fig. 2(d)]. The profiles in Fig. 2(d) can still be obtained through a phase transformation on the soliton shown in Fig. 2(c).

In addition to the one-peaked solutions, we also find solitons with more peaks originating from the complex Bloch modes at the lower edge of the first band [Fig. 2(e) and (f)]. They are similar to the multi-peaked gap solitons in a linear ${\mathscr{P}}{\mathscr{T}}$ lattice^30^ or the truncated-Bloch-wave solitons associating with the nonlinear Bloch waves in conventional (real) lattices^29^. Such solitons are composed of out-of-phase soliton components in neighboring lattice sites. Yet, the neighboring soliton components correspond alternatively to the nonlinear modes shown in Fig. 2(c) and (d) and respect opposite symmetries [Fig. 2(e) and (f)]. Overall, the real and imaginary parts of multi-peaked solitons hold the even and odd symmetries, respectively. Note that the amplitudes of soliton units are of the same value and are exactly equal to those of delocalized nonlinear Bloch waves at the same *b*. We should note that the amplitude of the imaginary part can be larger than that of the real part; see, e.g., Fig. 2(b) and (d). This only occurs when a system features an unbreakable ${\mathscr{P}}{\mathscr{T}}$ symmetry^34,35^.

**Figure 3:**
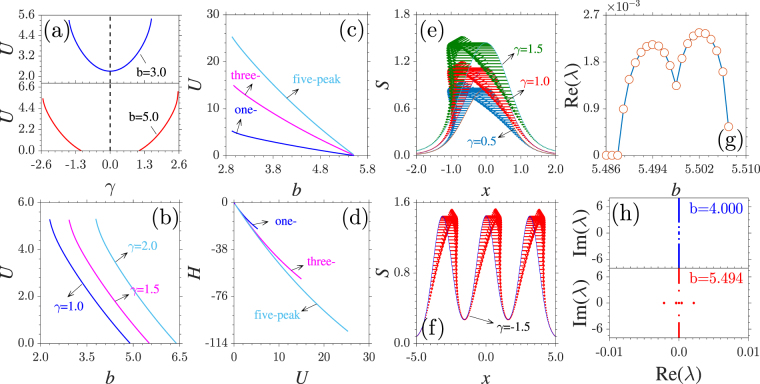
General properties of gap solitons. (**a**) Dependence of power *U* of one-peaked solitons at *b* = 3.0 (top) and 5.0 (bottom) on *γ*. (**b**) Power versus *b* for different *γ*. (**c**) Power of solitons with different numbers of peaks versus *b* at *γ* = 1.5. (**d**) Hamiltonian versus power corresponding to (**c**). (**e**) Transverse power-flow density of one-peaked solitons at different *γ*. (**f**) Transverse power-flow density of three-peaked soliton at *γ* =  −1.5. *b* = 4.0 in (**e,f**). (**g**) Instability growth rate Re(*λ*) versus *b* for three-peaked solitons at *γ* = 1.5. Notice the huge difference in scale between the horizontal axes in (**c**) and (**g**). (**h**) Stability spectrum for three-peaked solitons at *b* = 4.0 (top) and 5.494 (bottom).

At fixed chemical potential *b*, the dependence of the one-peaked soliton power *U* on the momentum coefficient *γ* is displayed in Fig. 3(a). The power curves are symmetric about *γ* = 0. With the growth of |*γ*|, the soliton power increases also in a parabolic manner. When *b* = 3.0, the existence domain of solitons occupies the whole first gap. However, one-peaked solitons at *b* = 5.0 exist only when |*γ*| > 1.1, which is exactly the lower edge of the first band. Meanwhile, *γ* = 2.6 corresponds to the upper edge of the second band.

Due to the repulsive nonlinearity, the power of solitons residing in one lattice site decreases monotonously with the growth of *b* [Fig. 3(b)]. With increasing *γ*, the power curve shifts towards the direction of the semi-infinite gap. This is because the existence domains of the solitons are always confined to the first bandgap of the corresponding linear system, which can be tuned by the momentum coefficient. Thus, tuning *γ* leads to the shift of the existence region of the solitons. At *γ* = 1.5, solitons with different numbers of peaks share the same existence domain [Fig. 3(c)]. At fixed *b*, the power of a five-peaked soliton is approximately five times the power of the single-peaked soliton. The Hamiltonian *H* of multi-peaked solitons decreases with increasing power *U*. The negative Hamiltonians guarantee the possibility for the existence of stable multi-peaked gap solitons [Fig. 3(d)].

For complex nonlinear modes, a transverse power-flow density in the form of $S=i/\mathrm{2[}\varphi (x){\varphi }_{x}^{\ast }(x)-{\varphi }^{\ast }(x){\varphi }_{x}(x)]$ may arise due to their nontrivial phase distribution^33^. The power-flow density curves of the single-peaked solitons at *γ* = 0.5, 1, and 1.5 are shown in Fig. 3(e). For *γ* 0, the energy always flows from right to left. The power-flow density is proportion to the modulus of the soliton. When *γ* < 0, the direction of the energy flow is reversed [Fig. 3(f)]. The unidirectional energy flow is in sharp contrast with that of a soliton in a conventional ${\mathscr{P}}{\mathscr{T}}$ lattice, where the direction of flow from gain to loss regions varies across the lattice channels^30,33^.

To elucidate the stability property of multi-peaked gap solitons, we search for perturbed solutions of Eq. (2) in the form of Ψ(*x*, *t*) = [*ϕ*(*x*) + *v*(*x*)exp(*λt*) + w^*^(*x*)exp(λ^*^t)]exp(*ibt*), where $v,w\ll 1$ are the infinitesimal perturbations, and *λ* is the complex growth rate of the disturbance. The linearization of Eq. (2) around *ϕ* yields a linear-stability eigenvalue problem:

5 $$i[\begin{array}{cc}{M}_{11} & {M}_{12}\\ {M}_{21} & {M}_{22}\end{array}]\,[\begin{array}{c}v\\ w\end{array}]=\lambda [\begin{array}{c}v\\ w\end{array}],$$

where ${M}_{11}=\frac{1}{2}\frac{{\partial }^{2}}{\partial {x}^{2}}+i\gamma \frac{\partial }{\partial x}+V+2g|\varphi {|}^{2}-b,{M}_{12}=g{\varphi }^{2}$, *M*_21_ = −(*gϕ*^2^)^*^, and ${M}_{22}=-{M}_{11}^{\ast }$. Nonlinear modes are stable if *λ* is purely imaginary or zero; otherwise, they are unstable.

According to Eqs (5), we conduct the linear stability analysis on all families of stationary solutions solved above. The results demonstrate that branches of solitons are almost completely stable in their entire existence domains. Taking three-peaked solitons as examples, the instability region (5.488, 5.507] occupies approximately 0.75% of the corresponding existence domain ([2.959, 5.507]) [Fig. 3(g)]. Even when *b* resides in the instability region, the growth rate is on the order of $\sim {10}^{-3}$. This implies that the unstable solitons are very robust and can remain unchangeable over a long period of time without obvious deformations. The detailed stability spectra of three-peaked solitons at *b* = 4.0 and 5.494 are illustrated in the top and bottom plots of Fig. 3(h).

**Figure 4:**
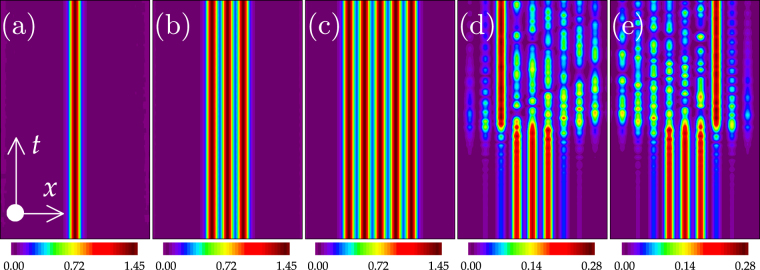
Evolution of different-peak-gap solitons. (**a**–**c**) Stable evolution examples of one-, three-, and five-peaked gap solitons corresponding to the solutions shown in Fig. 2(d–f). (**e**,**f**) Two unstable examples of three-peaked solitons at *γ* = +1.5 and −1.5. *b* = 4.0 in (**a–c**) and 5.494 in (**d**,**e**). *t* = 10000 in all the panels.

To verify the linear stability analysis results, we exhaustively simulate the evolution of solitons by the split-step Fourier method. Good agreements are obtained between the linear stability analysis and the direct numerical simulations. Several representative examples are displayed in Fig. 4. As expected, the weak instability occurs only when *b* approaches the upper edge of the first bandgap [Fig. 4(d,e)]. Unstable solitons are robust for a very long time without obvious distortions. The symmetric plots in Fig. 4(d,e) express again that the direction of the energy flow of solitons is determined solely by the sign of the momentum coefficient *γ*.

## Two-dimensional embedded solitons

Next, we discuss the formation of gap solitons in 2D periodic geometries. In this case, Eq. (2) becomes

6 $$i\frac{\partial \psi }{\partial t}=-\frac{1}{2}(\frac{{\partial }^{2}\psi }{\partial {x}^{2}}+\frac{{\partial }^{2}\psi }{\partial {y}^{2}})-i({\gamma }_{x}\frac{\partial \psi }{\partial x}+{\gamma }_{y}\frac{\partial \psi }{\partial y})-V(x,y)\psi -g|\psi {|}^{2}\psi \mathrm{.}$$

Here, *γ*_*x*_ and *γ*_*y*_ are the momentum coefficients in the *x* and *y* directions, respectively. We consider a quasi-1D potential in the form of $V(x,y)=6{cos}^{2}(x)exp(-{y}^{2}\mathrm{/4)}$, which is periodic along the *x* direction but localized along *y*^36,37^.

When *γ*_*x*_ = *γ*_*y*_ = 0, the linear system corresponding to Eq. (6) degenerates into the usual Hamiltonian operator *L*_0_*ϕ*_0_(*x*, *y*) = *b*_0_*ϕ*_0_(*x*, *y*). Applying the invertible transformation *ϕ*_0_(*x*, *y*) = *ϕ*(*x*, *y*)*exp*(*iγ*_*x*_*x* + *iγ*_*y*_*y*), one immediately finds that $b={b}_{0}+({\gamma }_{x}^{2}+{\gamma }_{y}^{2})/2$ also holds for all nonzero momentum coefficients, similar to the 1D case in the previous section.

**Figure 5:**
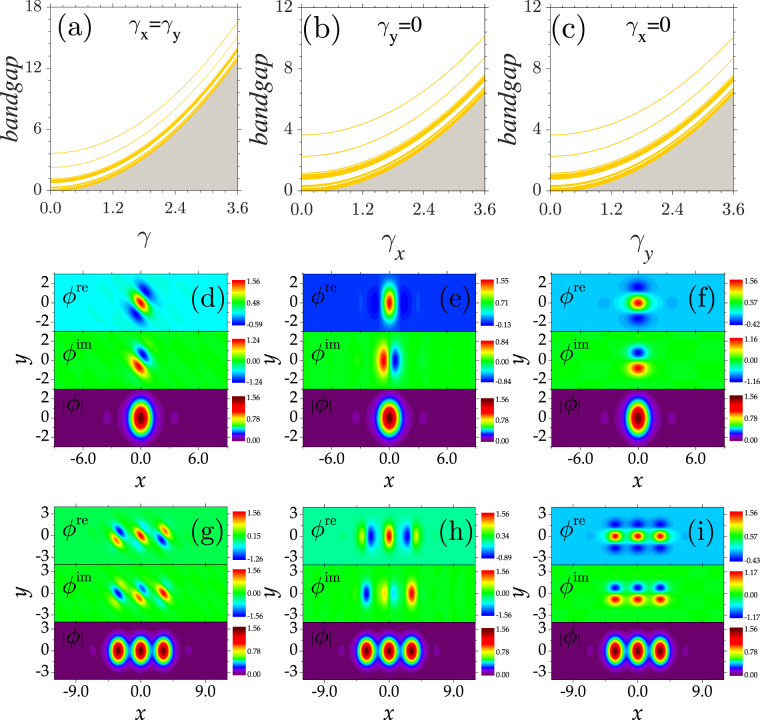
2D bandgap structures and soliton profiles. (**a**–**c**) Bandgap structures versus two transverse momentum coefficients *γ*_*x*,*y*_. (**d**–**f**) Profiles of one-peaked gap solitons. (**g**–**i**) Profiles of three-peaked gap solitons. *b* = 4.51 in (**d,g**) and 3.39 in (**e,f,h,i**). *γ*_*x*_ = *γ*_*y*_ = 1.5 in (**a,d,g**), *γ*_*x*_ = 1.5,*γ*_*y*_ = 0 in (**b,e,h**), and *γ*_*x*_ = 0,*γ*_*y*_ = 1.5 in (**c,f,i**).

One can see clearly from Fig. 5(a–c) that the bands are shifted toward the semi-infinite gap parabolically with increasing *γ*_*x*_ and *γ*_*y*_. For example, while the lower edge of the first band resides at *b* = 3.71 for *γ*_*x*_ = *γ*_*y*_ = 0 [Fig. 5(a)], the lower edge sits at *b* = 16.67 for *γ*_*x*_ = *γ*_*y*_ = 3.6. The corresponding *b* values at the lower band edges at *γ*_*x*_ = *γ*_*y*_ = 0 and *γ*_*x*_ = 3.6,*γ*_*y*_ = 0 are 3.71 and 10.19, respectively [Fig. 5(a,b)]. In other words, the relationships ${b}_{{\gamma }_{x}=3.6,{\gamma }_{y}=3.6}={b}_{{\gamma }_{x}=0,{\gamma }_{y}=0}+{\mathrm{(3.6}}^{2}+{3.6}^{2})/2$ and ${b}_{{\gamma }_{x}=3.6,{\gamma }_{y}=0}={b}_{{\gamma }_{x}=0,{\gamma }_{y}=0}+{\mathrm{(3.6}}^{2}+{0}^{2})/2$ hold for the 2D case. One can also expect that bands with different curvatures can be obtained for a fixed *γ*_*x*_ and varying *γ*_*y*_. Comparing with the 1D case, this offers a more flexible method for tuning the bandgap structure. Note that each band (gap) remains the same width for different *γ*_*x*, *y*_.

To illustrate the internal structure of 2D nonlinear solutions, we plot several representative examples of soliton profiles in Fig. 5(d–f). For *γ*_*x*_ = *γ*_*y*_ = 1.5, the system also respects a ${\mathscr{P}}{\mathscr{T}}$ symmetry. Yet, the symmetry axes of the real and imaginary parts of one-peaked solitons reside on the line of *y* = −*x* instead of on the *x* or *y* axis [Fig. 5(d)]. Concretely, the symmetry axis of the real profile is orthogonal to that of the imaginary profile, which guarantees the even symmetric distribution of the soliton modulus. The components of the one-peaked solitons satisfy the relations *ϕ*_*r*_(*x*,*y*) = *ϕ*_*r*_(−*x*, −*y*) and *ϕ*_*i*_(*x*,*y*) = −*ϕ*_*i*_(−*x*, −*y*). The symmetries of the present solitons are similar to those of solitons in inhomogeneous defocusing nonlinearities with a periodic gain and loss, where the ${\mathscr{P}}{\mathscr{T}}$ symmetry is shown to be unbreakable^35^.

For *γ*_*x*_ = 1.5 and *γ*_*y*_ = 0, the real part of a one-peaked soliton is even symmetric and the imaginary part is odd symmetric about *x* = 0. If *γ*_*x*_ = 0 and *γ*_*y*_ = 1.5, the distributions of the soliton components are orthogonal to those of solitons at *γ*_*x*_ = 1.5 and *γ*_*y*_ = 0. Although there exist slight differences between the real and imaginary parts shown in Fig. 5(e) and (f), their moduli are completely identical (see the bottom plots in the two figures). We note that such solitons originate from the corresponding complex Bloch modes at the lower edge of the first band. Therefore, their symmetries reflect the symmetric properties of the Bloch modes at different *γ*_*x*,*y*_. Similar to the 1D case, 2D solitons with asymmetric components can also be derived by applying a phase transformation to the symmetric components.

Solitons with more peaks exhibit similar symmetric properties to the one-peaked solitons [Fig. 5(g–i)]. If *γ*_*x*_ = *γ*_*y*_, the symmetry axes of each soliton component remain *y* = &minusplus;*x* [Fig. 5(g)]. On the whole, the relations *ϕ*_*r*_(*x*, *y*) = *ϕ*_*r*_(−*x*, −*y*) and *ϕ*_*i*_(*x*, *y*) = − *ϕ*_*i*_(−*x*, −*y*) are always satisfied. Additionally, the peak values of each soliton unit are equal. Despite the different distribution of the real and imaginary parts of the soliton profiles shown in Fig. 5(h) and (i), their moduli and power are completely identical to each other.

**Figure 6:**
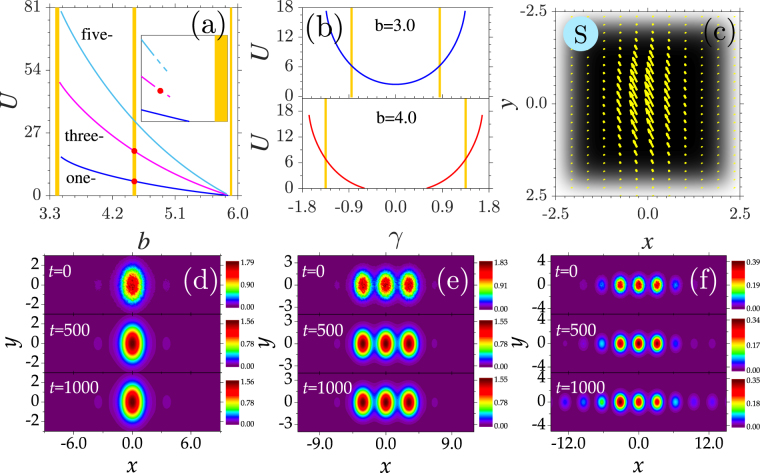
(**a**) Dependence of power *U* of one-, three-, and five-peaked solitons on *b*. Inset: Soliton power near the lower cutoffs. Solid: stable; dashed: unstable. Bands are shown in yellow and gaps in white. (**b**) Power versus *γ* for one-peaked solitons with different *b*. (**c**) Transverse power-flow density of one-peaked soliton. Evolution simulations of stable (**d**,**e**) and unstable (**//**) solitons marked in (**a**). *b* = 4.51 in (**c–e**) and 5.83 in (**f**). In all the panels, *γ*_*x*_ = *γ*_*y*_ = 1.5.

One striking finding in the present system with a quasi-1D potential and a momentum operator is that multi-peaked solitons exist in both the first and second finite gaps of the corresponding linear system, as well as the bandgap sandwiched between them [Fig. 6(a)]. In other words, such nonlinear modes can continuously cross the Bloch band associating with the continuous spectrum. According to refs^36,37^, stationary waves with *b* in the bands are the so-called “in-band” or “embedded” solitons. Unlike one-peaked solitons, multi-peaked solitons here have a threshold power, below which no stationary solutions can be found [inset plot in Fig. 6(a)]. Solitons cease to exist when *b* approaches an upper cutoff value near the lower edge of the second gap. The underlying physics of the existence of embedded solitons is that the symmetries of the real and imaginary linear Bloch modes in the second band are always opposite to those of nonlinear modes in the same region. Note that Bloch modes in the second band are *y*-antisymmetric, and multi-peaked solitons are *y*-symmetric. Thus, the resonance between Bloch modes and solitons cannot be excited. When *b* approaches the upper edge of the third band, the resonance between *y*-symmetric Bloch modes and solitons with the same symmetry prevent the solitons from penetrating into the third band.

At fixed chemical potential *b*, the power curves are symmetric about *γ*. The soliton power increases parabolically with the growth of *γ* [Fig. 6(b)]. Similar to the 1D situation, the variation in *γ* adjusts the bandgap structure of the system and thus the existence domain of the nonlinear modes. The penetration of *b* into the band and the shift of band with *γ* are also well presented in Fig. 6(b). The transverse power-flow density defined as $\overrightarrow{S}=i/2[\varphi \nabla {\varphi }^{\ast }-{\varphi }^{\ast }\nabla \varphi ]$ of the one-peaked embedded soliton at *b* = 4.51 is shown in Fig. 6(c). Due to the non-centrosymmetric potential, the direction of *S&vec;* does not coincide with the axis of *y* = −*x*.

Stability analysis results reveal that multi-peaked solitons are unstable only when *b* approaches the upper cutoffs. When one-peaked solitons are entirely stable in their whole existence domains, the instability region of three- and five-peaked solitons is only approximately 0.83% and 0.95% of their respective existence domains [inset plot in Fig. 6(a)]. One can expect that solitons with more peaks (e.g., 11,15) can be stable in wide parameter ranges. Direct numerical simulations of the stationary solutions verify the stability analysis results very well [Fig. 6(d–f)]. The strong noises added to the initial inputs radiate away quickly for stable solitons, and coherent bright patterns evolve invariably over arbitrarily long times. The unstable nonlinear mode suffers a symmetry breaking and becomes asymmetric and broader after a very long time [Fig. 6(f)].

## Conclusions

To summarize, we investigated two closely related problems. First, by introducing a momentum operator into the standard GPE, we realize the tunable bandgap structure of the system with a fixed periodic lattice. While the bands shift upward parabolically with the growth of the constant momentum coefficient, the width of each band and gap remains the same. For a nonzero momentum coefficient, the band edges are no longer symmetric about *k* = 0, which is in sharp contrast to the band spectrum of the conventional periodic systems. The nonzero momentum coefficient also leads to the existence of extended complex Bloch modes with asymmetric profiles, which can be derived by applying a phase transformation to the symmetric profiles. We showed that the ${\mathscr{P}}{\mathscr{T}}$ symmetry of the generalized GPE is never broken. Second, under repulsive interactions, multi-peaked matter-wave solitons bifurcating from the band edges were found in finite gaps. Such nonlinear localized modes are stable in almost their entire existence domain. We also demonstrated the existence of 2D complex truncated-Bloch-wave or embedded solitons supported by the generalized GPE. The in-band solitons originating from the edges of the Bloch bands exist in finite bandgaps of the corresponding linear system and continuously cross the Bloch band (continuous spectrum) sandwiched between (or neighboring) them. We anticipate that instead of a constant momentum coefficient, other forms of the momentum coefficient may provide more convenient or flexible methods for the realization of tunable bandgap structures.
